# Miscellany

**Published:** 1889-02

**Authors:** 


### MISCELLANY.

*The Naval Medical Examining Board*—Is now in session at the U. S. Naval Hospital, Philadelphia, for the examination of candidates for admission into the Medical Corps of the Navy as Assistant Surgeons.

The Board will remain in Philadelphia until the 31st of March, 1889.

After the 1st of April, 1889, the Board will hold its sessions at the Naval Hospital, Brooklyn, N. Y.

There are fourteen vacancies in the grade of Assistant Surgeon.

Further information may be obtained by addressing the President of the Examining Board. J. MILLS BROWNE,
*Surgeon General, U. S. Navy.*

NAVY DEPARTMENT,
BUREAU OF MEDICINE AND SURGERY,
*Washington, D. C., Jan. 16, 1889.*

---

***Circular for the Information of Persons Desiring to Enter the Medical Corps of the U. S. Navy.***—A candidate for examination and appointment in the Medical Corps of the Navy must be between 21 and 26 years of age, and must apply to the Honorable Secretary of the Navy for permission to appear before the Naval Medical Examining Board.

The application must be in the handwriting of the applicant, stating age and place of birth, also, the place and State of which he is a permanent resident; and must be accompanied by letters or certificates from persons of repute, testifying, from personal knowledge, to his good habits and moral character, and that he is a citizen of the United States.

FORM OF APPLICATION.

————————,
————, 188

TO THE HONORABLE
SECRETARY OF THE NAVY,
*Navy Department, Washington, D. C.:*

SIR: I request permission to be examined for an appointment as Assistant Surgeon in the United States Navy.

I was born at ——, and was —— years of age on the —— day of ——, 188—, and am a citizen of the United States, residing in ——, county of ——, in the State of——.

I enclose herewith certificates as to moral character, habits and citizenship.

Very respectfully,

———— ————

If, in reply to the above, the candidate receive a permit, he will notify the President of the Board of the fact, and request him to appoint a time for his examination.

Candidates will be expected to present to the Board testimonials of educational and professional fitness.

The Board is required, under oath, to report on the physical, mental, moral and professional qualifications of the candidate; so that the examinations are necessarily rigid and comprehensive, though simple and practical, and not beyond the attainments of any well-educated physician.

A successful candidate, upon the completion of his examination, will be notified by the President of the Board that he has been found qualified.

An applicant found "not qualified" may be allowed a second examination after one year, but not a third.

No allowance will be made for the expenses of persons undergoing examination, which, if uninterrupted, is usually completed within a week.

Appointments will be made as vacancies occur, and in the order of merit reported by the Board, but a qualified candidate, not appointed within a year, must be re-examined.

The officers of the Medical Staff of the Navy are as follows: Medical Directors, Medical Inspectors, Surgeons, Passed Assistant Surgeons and Assistant Surgeons.

Vacancies in these grades (by death, or retirement at the age of sixty-two years) are filled in the order of seniority, and for each step of promotion a physical and professional examination is required by law.

The pay of an Assistant Surgeon in the Navy is $1,000 per annum "on leave or waiting orders," $1,400 "on shore duty," $1,700 "at sea," and, when at sea, one ration at 30 cents per diem in addition.

Eight cents a mile is the allowance when traveling under orders.

ORDER OF EXAMINATION.

1. Physical. 2. Written. 3. Oral.
4. Clinical. 5. Practical.

*The Physical examination* will be very thorough, and the candidate will be required to certify, under oath, that he is free from any mental, physical or constitutional defects, hereditary taint, or disability of any kind that would be liable to interfere with the efficient performance of duty.

*Written examination.*—The candidate will be required to address a letter to the Board of Examiners, stating concisely—

1. The date and place of his birth; the school, institution, or college at which he received his general education; the several branches studied, including his knowledge of general literature, and of the ancient and modern languages; the exact title of the medical school or schools at which he received instruction, and, if an Alumnus, the date of his graduation; the name and place of residence of his preceptor, and the time when he commenced the study of medicine; also, the title of the text-books studied or read on Chemistry, Anatomy, Physiology, Histology, Materia Medica, Pharmacy, Therapeutics, Theory and Practice of Medicine, Principles and Practice of Surgery, Minor Surgery or Mechanical Therapeutics, Medical Jurisprudence, Toxicology, Obstetrics, Hygiene, Biology and Physics.

2. The opportunities he has had of engaging in the practice of medicine, surgery and obstetrics, or of receiving clinical instruction; or whether he has or has not been a resident physician or interne in a civil or military hospital.

3. The number of subjects or parts of subjects he has dissected; what opportunity he has had to become familiar with minor surgery and bandaging, chemical and pharmaceutical manipulations, and the physical properties of drugs.

The candidate will append to this letter his name in full, post-office address, and his local address at the date of the examination.

*A thesis or short essay* must next be written (without reference to notes or books) upon some professional or scientific subject indicated by the Board.

Written answers will then be required to twelve or more questions, propounded by the Board, on the following subjects:

Anatomy, Histology, Physiology, Surgery, Theory and Practice of Medicine, Obstetrics, Materia Medica, Chemistry, Hygiene, Medical Jurisprudence, Toxicology and Physics.

*Oral examination.*—The candidate will be examined orally upon his literary and scientific acquirements, including general history, natural science and English literature, and professionally upon Anatomy, (general, special and surgical), Histology, Physiology, Theory and Practice of Medicine, Principles and Practice of Surgery, Chemistry, Legal Medicine, Toxicology, Materia Medica, Therapeutics, Pharmacy, Obstetrics and Diseases of Women and Children, Hygiene, Microscopy and Physics.

Candidates possessing special knowledge of the higher Mathematics, Astronomy, Geology, Botany, Zoology, Literature, Art and Ancient and Modern Languages, will be given full credit for their proficiency.

*The Clinical examination of patients* will be made by the candidate at a Naval Hospital, and will include the use of the Microscope, Thermometer, Laryngoscope, Ophthalmoscope and other aids to physical diagnosis; after which he will be required to submit a written clinical report on one or more medical or surgical cases.

*The practical examination* will comprise surgical operation on the cadaver, the application of splints, bandages and surgical dressings, the use of the microscope (for clinical purposes, and the recognition of pathological or other specimens) and chemical and pharmaceutical manipulations.

A candidate may withdraw at any period of the examination, with the consent of the Board, and may at a future time present himself for re-examination.

The Board may conclude the examination —written, oral or practical—at any time, and may deviate from this general plan in such manner as it may deem best to insure the interests of the naval service.

William C. Whitney,
*Secretary of the Navy.*

---

***Illinois State Board of Health.***—Since the Board has been somewhat roundly abused, and misrepresented, by some of the newspapers in Illinois and other states, the following, taken from the recent message of (now) Ex-Governor Richard J. Oglesby, is of no little interest:

"The intelligent and faithful discharge of the duties imposed by law upon the State Board of Health, and the benefits which accrue therefrom to the commonwealth, sufficiently attest the wisdom of the Legislature in the creation of this organization. From the vast field covered by its labors it is only possible, in this connection, to single out for mention a few of its most important works. It is charged by the constituting act with the supervision of the interests of the life and health of the citizens of the State, and to this end, the Board has addressed its efforts more particularly to the limitation and—so far as is practicable—to the prevention of epidemics of contagious and infectious diseases.

"It is a matter of record—a fact which I understand has now passed into the authentic history of epidemics in this country—that the labors of the Board in this direction resulted in a saving of nearly $3,500,000 to the people of the State in 1881 and 1882, when small-pox was epidemic. Through the preventive and protective measures then established and since enforced, there has been no repetition of that disease in an epidemic form.

"The wise and intelligent policy of the Board on the subject of quarantine has been of great value to the material interests, not only of Illinois, but of the whole Mississippi Valley. While vigilantly guarding against the introduction and spread of the dangerous, contagious and infectious diseases, it secures the least interference with commerce and travel, and so averts unfounded panics and prevents loss and interruption of business and industry. During the past few months a striking illustration of the value of this policy was afforded by the action of the worthy Secretary of the Board, who refused to sanction any expenditure of money from the public treasury in the maintenance of quarantine restrictions, which his wide and varied experience and scientific knowledge enabled him to pronounce unnecessary for the State. His firmness in this instance alone prevented the loss of thousands of dollars, besides great inconvenience to travelers and vexatious interference with business; and the example thus set materially helped to check the ruinous and needless quarantine enforced in other States."

The Governor then speaks of the sanitary survey of the State, begun in 1883 and still in progress; of the careful analyses now being made of the water-supply of the State; and of the benefits that have accrued from the enforcement of the Medical Practice Act.

---

***Consumptives and Long Sea Voyages.***—Dr. J. De Burgh Griffith, Physician to Melbourne Hospital, says in *The British Medicol Journol* of February 9th: It is customary with English and Irish physicians to recommend long sea voyages to phthisical patients, and frequently prescribe a voyage by sailing vessel to Australia. In theory this is excellent advice—ozone, sea air, bright sky, warm weather all the voyage; but in practice it is very different. My personal experience of sea voyages and my position at the Antipodes enable me to speak with some knowledge of the effects of a sailing-ship voyage on delicate people, and I feel quite sure that medical men do not know what they are advising when they recommend patients in advanced phthisis to come to Australia in a sailing ship.

Land folks appear to think that an Australian voyage is all sunshine and warm weather. This may be true of part of the passage; but it is a small part. The time in the English Channel alone is often enough to murder any but seasoned seamen; then in "running down the Easting," in lat. 39°, 40°, 41° South, cold, dark, wintry weather and snow are frequently met with. The food is inferior, fresh vegetables are not procurable, and there are usually no comforts sufficient for delicate people—no saloon fire, no hot water at night, and, if clothing gets wet, there is no means of drying it except on deck.

In a well-appointed passenger steamship all this is quite different. If delicate people are ordered to Australia, I venture to suggest that a more careful selection of cases should be made, and none sent out who are far advanced in phthisis, or who do not present a fair chance of recovery. Patients with serious lung disease and a rapid downward tendency should never be sent from home. It is much more merciful to allow these poor things to die amongst their own people than to be sent abroad to die with strangers in a strange land.

For a certain clase of phthisical people I have no hesitation in recommending a sea voyage and residence in Australia; but if any good is to be derived, the passage must be made in a well appointed steamship and not in a sailing cargo vessel. There is plenty of choice in either case. Choose some of the grand steamships trading regularly with Australia. If it be summer time, or the hot season in the Red Sea, I recommend the Cape route direct to Melbourne.

---

*Royal Academie of Sciences of Turin. The Bressa Prize.*—The Royal Academy of Sciences of Turin, in accordance with the last will and testament of Dr. Cesare Alessandro Bressa, and in conformity with the programme published December 7th, 1876, announces that the term for competition for scientific works and discoveries made in the four previous years 1885-88, to which only Italian authors and inventors were entitled, was closed on December 31st, 1888.

The academy now gives notice that from the 1st of January, 1887, the new term for competition for the seventh Bressa prize has begun, to which, according to the testator's will, scientific men and inventors of all nations will be admitted. A prize will therefore be given to the scientific author or inventor, whatever be his nationality, who during the years 1887-90, "according to the judgment of the Royal Academy of Sciences of Turin, shall have made the most important and useful discovery, or published the most valuable work on physical and experimental science, natural history, mathematics, chemistry, physiology and pathology, as well as geology, history, geography and statistics."

The term will be closed at the end of December, 1890.

The value of the prize amounts to 12,000 Italian lire.

The prize will in no case be given to any of the National members of the Academy of Turin, resident or non-resident.—*A. Genocchi, President of the Royal Academy; A. Naccari, Secretary of the committee.*

*Turin*, January 1st, 1889.

---

The Director of the Statistical Bureau of the Hungarian Academy of Sciences states that from his researches in the statistics of 24,000 cases he draws the following conclusions: Children of fathers between 25 and 40 years of age are the healthiest, those of fathers under 25 are weak, and those of fathers over 40 are weaker. In regard to the mothers: Children born before the 35th year of the mother's life are strongest; those born between the 35th and 40th year of the mother's life are about 8 per cent., and those born after the mother is 40 are about 10 per cent., weaker. Children of parents near the same age are weaker than children of older fathers and younger mothers. But if a woman has already arrived at from 30 to 35 years, it is better for the children that she marry a man between 20 and 30.

---

*Shock in Relation to Dental Operations.*—This subject, which has received but little attention, though of considerable importance, was brought forward by Dr. Trueman in an able paper at the Odontological Society of Pennsylvania at the November meeting. Under this heading he included not merely the collapse which may follow an operation or extreme emotion, but the lesser yet more prolonged depression of the mental powers and the circulatory system. To illustrate the importance of the matter he quotes a case occurring in Dr. Black's practice. A young lady at her own urgent request had three hours' continuous operation, some part of which was intensely painful. She was then dismissed for a couple of hours and returned promptly. After about another hour evidences of collapse appeared and the operations were suspended. On the route home she became worse, and remained in a "stupid condition" for four or five days, after which she passed into a nervous fever for several months. This is doubtless an exceptional and extreme case; but all dentists know of cases, by no means infrequent, of persons exhibiting great weakness and depression after prolonged dental operations. The craze for enormous gold operations, taking five or six hours' continuous sitting, has, Dr. Trueman hopes, passed by, and he advocates that an operation should not last more than two hours, and that an interval of several days should elapse before another is undertaken, so as to minimize as much as possible the effects of the resulting nervous strain. Referring to the shock caused by extractions, he says the shortness of the operation may lead some to regard it as of little moment. A few favored individuals will sit down and have a tooth drawn with the greatest equanimity, but as a rule there is great mental strain in advance, and great shock during the operation, and he condemns the practice of removing a large number of teeth at one sitting. Discussing the profession of dentistry as a calling, he considers it one of the most trying. Standing for hours, it is an unbroken effort of the mental and physical powers of the operator in almost constant conflict with similar disturbed relations of the patient; meeting all sorts and conditions of men, women and children—the women all nerves, the men all impatience, and the children all frightened,—it is not to be wondered at that many fall away from the ranks in comparatively early life. This is perhaps rather a gloomy view of the matter, but there is a great deal of truth in it.—*Lancet*, Feb. 9, 1889.

---

*Salicylic Acid as a Diuretic.*—After a series of investigations on this subject, Huber concludes that salicylic acid is one of the safest and most important diuretics. The greatest increase in the amount of urine seems to occur in rheumatic fever and serous pleurisy, whether the temperature is raised or not. In all cases the total loss of water by the skin and urine was increased, and the solids of the urine were increased. In ordinary pleurisy, and in four cases of cardiac dropsy the drug acted well.

---

*Effect of Tobacco-Smoke on Micro-Organisms.*—Dr. Vicenzo Tassinari, assistant at the hygienic institute of the University of Pisa, publishes some interesting experiments with tobacco smoke on various pathogenic and non-pathogenic organisms. The duration of the fumigations varied from thirty to thirty-five minutes, and the quantity of tobacco consumed amounted to from $3\frac{1}{2}$ to $4\frac{1}{2}$ grammes. It was shown that tobacco smoke possesses the property of retarding the development of some pathogenic bacteria and preventing the growth of others. Thus the smoke from a large Virginia cigar retarded the development of the bacterium pradigiosus for seventy-two hours, of the staphylococcus aureus for seventy-three hours, and of the anthrax bacillus for ninety-seven hours. No development of colonies of the spirillæ of Asiatic cholera and anthrax, and of the bacilli of typhus and pneumonia was observed after from a hundred and twenty-eight to a hundred and sixty-eight hours. The author regards these results as due to the chemical action of the ingredients of tobacco-smoke.

***A Bullet in the Chest for Thirteen Years.***—Dr. R. C. Macdonald recently removed a rifle bullet from a man which had been in the chest for thirteen years. The case is chiefly remarkable from the fact that the bullet had remained in the pleural cavity for so long a time without setting up pleurisy. It entered the back of the shoulder immediately below the spine of the scapula, and probably entered the pleural cavity by penetrating the intercostal muscles. The operation was performed with antiseptic precautions, and the wound soon healed. A splinter had been removed from the wound of entrance some months after the accident, which occurred to the man whilst marking at a rifle range in Beith. The bullet had gravitated to the lower part of the chest.—*The Lancet.*

***Medical and Surgical Register of the United States.***—Messrs. R. L. Polk and Company, publishers of the "Medical and Surgical Register of the United States," annouuce that they have now in preparation the second edition of the "Register," which affords a convenient opportunity to the medical profession of the country to secure the best directory of the physicians of the United States that has ever been published, and with it much valuable information concerning medical matters, not given elsewhere in any single work. The publishers have offices in Detroit, Chicago, Baltimore, Atlanta, St. Paul, Minneapolis, Indianapolis, St. Louis, Toronto, and Portland, Oregon.

***For What are Doctors Paid?***—An English judge has recently given a decision containing a great deal of common sense. A local surgeon sued the executor of a deceased farmer for an amount for consultations. The deceased could not take any medicine. The judge, in giving his decision, said: "Many people of a better class hold the idea that it is medicine doctors are paid for. It is for skill."

***Treatment of Hysterical Vomiting.***—Ewald recommends the following:

Hydrochlorate of morphine . 0.20 centig.
Hydrochlorate of cocaine . . . 0.30 centig.
Tincture of belladonna . . . . . 5 grains.
Cherry-laurel water . . . . . . 25 grains.

S. Take 10 or 15 drops every hour, till the vomiting ceases.

***Dr. Emile Dubois-Reymond*** celebrated his 70th birthday on November 7th. He is the son of a watchmaker, and began his scientific studies at Bonn in 1838. Johannes Müller first gave his thoughts the direction in which he has done so much brilliant work, and whén Müller died he was succeeded in the chair of Physiology at Berlin by Dubois-Reymond.

***Blisters in Heart-Disease.***—Jacond has recently called attention to the dangers of blistering in cardiac disease in cases in which the urine contains albumen, even the smallest trace. Cantharides should be absolutely proscribed in these cases. and iodine used if such counter-irritation be thought necessary.

***Animal Vaccination in France.***—The French Home Minister has presented 8,000 francs ($1,600) to the Académie de Médecine, for the encouragement of animal vaccination.

